# Association Between Methane Concentration Measured by a Modified Mask System and GreenFeed Methane Production in Dairy Cows

**DOI:** 10.3390/ani16193156

**Published:** 2026-10-08

**Authors:** Marta Sfulcini, Vincenzo Lopreiato, Fiorenzo Piccioli-Cappelli, Erminio Trevisi, Andrea Minuti

**Affiliations:** 1 Department of Animal Science, Food and Nutrition (DIANA), Faculty of Agricultural, Food and Environmental Sciences, Università Cattolica del Sacro Cuore, 29122 Piacenza, Italy; marta.sfulcini@unicatt.it (M.S.); fiorenzo.piccioli@unicatt.it (F.P.-C.); erminio.trevisi@unicatt.it (E.T.); 2 Department of Veterinary Sciences, Università di Messina, 98168 Messina, Italy; vincenzo.lopreiato@unime.it; 3 Romeo and Enrica Invernizzi Research Center for Sustainable Dairy Production of the Università Cattolica del Sacro Cuore (CREI), 29122 Piacenza, Italy

**Keywords:** enteric methane, dairy cows, methane phenotyping, GreenFeed, breath methane concentration

## Abstract

Accurate measurement of enteric methane (CH_4_) emissions is essential for methane phenotyping, but established techniques may be costly and difficult to apply to large numbers of animals. This study evaluated a simple mask-based system for measuring CH_4_ concentration in exhaled breath of lactating dairy cows in comparison with CH_4_ production measured using GreenFeed. Thirty-two Holstein cows were evaluated, generating 60 paired observations. On each sampling day, exhaled breath was collected using the mask during three 5 min periods, and CH_4_ concentration was determined by micro gas chromatography. Mask CH_4_ concentration was positively associated with GreenFeed CH_4_ production (Pearson r = 0.616; Spearman ρ = 0.606; *p* < 0.001). The association remained significant after accounting for repeated observations within cows using a linear mixed-effects model (β = 53.1 ± 15.7 g/day per 0.1 percentage-point increase in mask CH_4_, 95% CI: 22.3 to 84.0; *p* < 0.001). An exploratory within-cow analysis was also performed in four cows evaluated over five sampling days. Positive within-cow correlations were observed in all four animals (r = 0.157–0.699), but none reached statistical significance (*p* > 0.05). These results indicate that CH_4_ concentration measured using the mask contains information on differences in methane emission among cows and may have potential as a methane phenotyping measure.

## Simple Summary

Reducing methane emissions from dairy cattle requires practical methods to identify animals that naturally produce more or less methane. Current methods for measuring methane emissions can be expensive and require specialized equipment, which limits the number of animals that can be evaluated. In this study, we tested a simple and portable mask system that collects a small portion of the air exhaled by dairy cows. Methane concentration measured with the mask was compared with methane production measured using an established automated system that records emissions while cows voluntarily visit a feeding station. Measurements obtained with the two systems were positively related, and cows with higher methane concentrations measured by the mask generally also had higher methane emissions measured by the automated system. An exploratory analysis of repeated measurements in four cows showed positive but non-significant relationships between the two measurements; therefore, further studies are needed to determine whether the mask can track short-term changes within individual cows. The mask is not intended to provide an exact estimate of daily methane production, but could represent a simple and relatively inexpensive method for methane phenotyping in dairy cows.

## 1. Introduction

Enteric methane (CH_4_) produced during ruminal fermentation is an important contributor to greenhouse gas emissions from livestock and represents a loss of dietary energy that could otherwise be utilized by the animal [1,2]. Accurate measurement of enteric CH_4_ emissions is therefore essential to evaluate mitigation strategies, improve emission inventories, and support research aimed at understanding the biological factors associated with methane production.

Several techniques have been developed to quantify enteric CH_4_ emissions in ruminants, including respiration chambers (RC), the sulfur hexafluoride (SF_6_) tracer technique, and, more recently, the GreenFeed (GF) system [3]. RC are generally considered the reference method because of their high accuracy and precision [4,5]. However, their application is limited by high installation and operating costs, low experimental throughput, and the potential to alter normal animal behavior and feed intake. Alternative approaches, including the SF_6_ tracer technique [6] and the GF system, have reduced some of these limitations by allowing methane measurements under less restrictive conditions. Nevertheless, these methods still require specialized equipment, dedicated infrastructure, and considerable investment [7]. Consequently, there is growing interest in developing alternative approaches that are simpler, less expensive, and suitable for evaluating larger numbers of animals under practical conditions. In this context, measurements of CH_4_ concentration in exhaled breath have been proposed as an alternative phenotype for discriminating animals according to their methane emissions. Previous studies using sniffers or laser-based systems have shown associations between breath CH_4_ concentration and CH_4_ production measured using established techniques, supporting the potential use of concentration-based measurements for ranking animals [8,9,10].

Among short-term sampling techniques, face-mask systems have also emerged as a promising approach for collecting exhaled gases from cattle and have shown encouraging results when compared with established methane measurement techniques [11,12]. However, information regarding the performance of simple mask-based systems compared with established methane measurement techniques remains limited.

To address the need for a practical methane measurement system, we developed a simple mask-based device for collecting exhaled methane from dairy cows. Compared with currently available techniques, the proposed system is portable, relatively low-cost, and easy to implement, making it potentially suitable for studies involving a large number of animals. Before such a system can be adopted in research applications, however, its performance should be evaluated against an established reference method.

Therefore, the objectives of the present study were to evaluate the association between CH_4_ concentration measured with the mask system and CH_4_ production measured with GreenFeed, with particular emphasis on the ability of the mask system to discriminate animals according to their relative methane phenotype, and to explore whether temporal changes in mask CH_4_ concentration reflected corresponding changes in GreenFeed CH_4_ production in a subset of cows repeatedly evaluated over consecutive days.

## 2. Materials and Methods

### 2.1. Animals and Study Design

The study was conducted between November and December 2025 at the CERZOO experimental dairy farm of Università Cattolica del Sacro Cuore (San Bonico, Piacenza, Italy). The study included 32 lactating Holstein dairy cows, of which 9 were primiparous and 23 were multiparous. Descriptive characteristics of the animals, including age, parity, days in milk (DIM), body weight (BW), body condition score (BCS), milk yield, and dry matter intake (DMI), are reported in Table 1.

Cows ranged from 47 to 321 DIM and from first to fifth lactation. Throughout the experiment, all cows were housed together in a single free-stall pen, received the same total mixed ration formulated to meet or exceed the nutrient requirements of lactating dairy cows ([13]), and had ad libitum access to water. The ingredient and chemical composition of the diet are reported in Table 2.

Two GreenFeed emission monitoring systems (C-Lock Inc., Rapid City, SD, USA) were installed within the pen, allowing unrestricted voluntary access to all animals throughout the study.

The study comprised two complementary components. First, CH_4_ concentration measured using a modified mask-based sampling system was compared with CH_4_ production recorded by GreenFeed across animals. Second, four cows were repeatedly evaluated over five consecutive days to explore whether changes in mask CH_4_ concentration across days reflected corresponding changes in GreenFeed CH_4_ production within the same animal. Overall, 60 cow-day observations were available for analysis. For each cow-day observation, mask measurements were obtained from three 5 min collections performed at different times of the day, as described below.

### 2.2. Methane Sampling and Measurement

Methane concentration in exhaled breath was measured using a modified mask-based sampling system (Figure 1).

Overall, 60 cow-day observations were obtained from 32 cows: four cows contributed five observations each, 12 cows contributed two observations each, collected approximately 7–10 days apart; and the remaining 16 cows contributed one observation each. The system was developed from a commercially available equine inhalation mask (AirOne Flex X-Full, Albert Kerbl GmbH, Buchbach, Germany) modified to separate inspiratory and expiratory airflow. During inspiration, ambient air entered the mask through the lateral valves, whereas during expiration, the lateral valves closed and exhaled air was directed through the anterior expiratory valve. The mask was secured to the animal’s head using the adjustable hook-and-loop fastening straps supplied by the manufacturer.

A sampling port located immediately upstream of the expiratory valve was connected through inert tubing to a peristaltic roller pump (G928, FTVOGUE, Shenzhen, China), operated at a constant sampling flow rate of 200 mL/min, and to a custom-fitted 2 L multilayer high-barrier pouch (PET/ALU/PET/PP; ST3T.0645, Goglio S.p.A., Daverio, Italy) used for gas collection. During expiration, the pump continuously diverted a fraction of the exhaled air into the sampling pouch, whereas the remaining expired air was released to the environment through the expiratory valve. Prior to sampling, bags were completely evacuated using a pump. After Micro-GC analysis, bags were flushed three times with atmospheric air, fully evacuated, and sealed until subsequent use.

On each sampling day, three separate 5 min mask collections were performed for each cow, at approximately 8:00, 14:00, and 18:00 h. Cows were milked twice daily at approximately 5:00 and 17:00 h, and fresh TMR was delivered once daily at approximately 8:30 h. Sampling times were selected to obtain measurements at different times throughout the day and at different intervals relative to feeding and milking, while remaining compatible with the routine management of the herd. A separate gas sampling bag was used for each collection, resulting in three gas samples per cow per sampling day. Before each collection, the mask was correctly positioned and secured to the animal’s head. The mask fit was visually checked to ensure that the flexible sleeve adhered closely around the muzzle, minimizing the entry of ambient air. The cow was also visually monitored for signs of discomfort or altered breathing. At the end of the 5 min collection, the corresponding gas sampling bag was disconnected, sealed, labelled, and transported to the laboratory for analysis. Within-day variability of mask CH_4_ concentration was assessed by calculating, for each cow-day observation, the coefficient of variation (CV) among the three 5 min collections obtained from the same cow on that sampling day.

Methane concentration in each gas sample was determined within 2 h of collection using a 490 Micro GC gas chromatograph (Agilent Technologies, Santa Clara, CA, USA) equipped with a COX BF column and a micro thermal conductivity detector (μTCD). Helium was used as the carrier gas. The injection time was 20 ms, the column temperature was maintained at 80 °C, and the total analysis time was 190 s. Gas components were identified by comparing their retention times with those obtained after separate injections of reference gases of known identity. Methane was quantified by external calibration using pure nitrogen as a zero-CH_4_ reference (0.0% CH_4_) and certified CH_4_ gas mixtures (SAPIO Produzione Idrogeno Ossigeno S.r.l., Milano, Italy) at concentrations of 526 ppm (0.0526%) and 5070 ppm (0.5070%). A three-point calibration curve was generated by linear regression of the CH_4_ chromatographic peak area against the corresponding CH_4_ concentration. The calibration showed good linearity over the investigated concentration range (R^2^ = 0.99), and the CH_4_ concentration in each sample was calculated from the resulting regression equation. Gas samples were introduced by directly connecting the sampling bag to the instrument injection system through a stainless-steel valve, without sample pretreatment. Each gas sample was analyzed three consecutive times by Micro GC as analytical replicates, and CH_4_ concentration was expressed as the percentage of CH_4_ in the sampled gas.

Methane production was also monitored using two GreenFeed emission monitoring systems (C-Lock Inc., Rapid City, SD, USA) installed in the free-stall barn. Cows were individually identified by radio-frequency identification ear tags and had voluntary access to both units throughout the study. Pelletized bait feed was provided as an attractant during GreenFeed visits. Each cow was allowed a maximum of six rewarded visits per day, with a minimum interval of 3 h between visits and a maximum of eight feed drops (35.94 ± 1.96 g/drop) per visit. The bait pellet used in the GreenFeed units was Tekno Robot CREI (Ferrero Mangimi S.p.A., Farigliano, Cuneo, Italy). Mean bait pellet intake during the study was 1.11 ± 0.27 kg/cow per day, and its composition is reported in Table 2.

The GreenFeed units were calibrated weekly, and CO_2_ recovery tests were performed monthly according to the manufacturer’s recommendations. Mean CO_2_ recovery was 104.08% and 102.6% for the two units, respectively. Methane production was quantified during individual visits using the manufacturer’s acquisition and quality-control algorithms, and only visits meeting the quality criteria established by the GreenFeed software were retained for analysis.

The average number of valid GreenFeed visits was 3.3 ± 1.6 visits per cow per day, and the average duration of valid data collection during each visit was 267 ± 65 s.

### 2.3. Data Processing

For each 5 min mask collection, methane concentration was calculated as the arithmetic mean of the three consecutive Micro GC analytical determinations performed on the corresponding gas sample. The daily mask value for each sampling day was then calculated as the arithmetic mean of the three 5 min mask collections performed at approximately 8:00, 14:00, and 18:00 h.

GreenFeed methane production was expressed as grams of CH_4_ per day. An exploratory analysis was conducted to evaluate the temporal relationship between mask CH_4_ concentration and GreenFeed CH_4_ production. Different combinations of GreenFeed daily CH_4_ production were evaluated within a 5 d period centered around each mask sampling day, from 2 days before (Day −2) to 2 days after (Day +2). Associations with daily mask CH_4_ concentration were evaluated using GreenFeed CH_4_ production from each individual day (Days −2, −1, 0, +1, and +2) and from three contiguous 3 d averaging windows (Days −2 to 0, Days −1 to +1, and Days 0 to +2). GreenFeed windows including days following mask sampling were interpreted as estimates of short-term CH_4_ production rather than as contemporaneous measurements. Pearson and Spearman correlation coefficients were calculated for each temporal window.

The temporal window selected on the basis of these comparisons was subsequently used to define the GreenFeed CH_4_ production value associated with each mask sampling day. Each daily mask CH_4_ concentration was paired with the corresponding GreenFeed CH_4_ production value derived from the selected temporal window, generating a dataset of 60 paired observations.

The complete dataset of 60 paired observations was used to evaluate the overall association between mask CH_4_ concentration and GreenFeed CH_4_ production. Because some cows contributed observations on more than one sampling day, cow identity was retained in the dataset and accounted for in the statistical analysis. To specifically evaluate the association between the two measurement approaches at the cow level, an additional analysis was performed using one observation per cow. For each of the 32 cows, mask CH_4_ concentration and the corresponding GreenFeed CH_4_ production (Days 0 to +2) were averaged across all available sampling occasions. Pearson and Spearman correlation coefficients were then calculated using these 32 cow-level mean observations.

In addition, four cows with repeated measurements over five sampling days were considered separately in an exploratory within-cow analysis. For each sampling day, the daily mask CH_4_ concentration, calculated as the mean of the three 5 min collections, was paired with the corresponding GreenFeed CH_4_ production averaged over Days 0 to +2, to evaluate whether changes in mask CH_4_ concentration across sampling days were associated with corresponding changes in GreenFeed CH_4_ production.

### 2.4. Statistical Analysis

Statistical analyses were performed using SAS software (version 9.4; SAS Institute Inc., Cary, NC, USA). Statistical significance was declared at *p* ≤ 0.05, whereas 0.05 < *p* ≤ 0.10 was considered a statistical tendency.

The overall association between methane concentration measured with the mask system and methane production recorded by the GreenFeed system was evaluated using Pearson’s correlation coefficient, while Spearman’s rank correlation coefficient was used to evaluate the rank-based association between the two measurements. These correlations were first calculated using the complete dataset of 60 paired cow-day observations. A cow-level analysis was also performed using the 32 cow-level mean observations described above. Pearson’s and Spearman’s correlation coefficients were calculated to evaluate the linear and rank-based associations, respectively, between mean mask CH_4_ concentration and mean GreenFeed CH_4_ production across cows.

Because the dataset included repeated observations from a subset of cows, a linear mixed-effects model was additionally fitted to evaluate the association between mask CH_4_ concentration and GreenFeed CH_4_ production while accounting for repeated observations within cows. GreenFeed CH_4_ production was included as the dependent variable, mask CH_4_ concentration as a fixed effect, and cow as a random intercept. Residuals were assumed to be independently and normally distributed.

Finally, the ability of the mask system to detect temporal variation in methane emissions within individual animals was explored using the subset of repeatedly sampled cows (*n* = 4). For each cow, Pearson’s correlation coefficient and Spearman’s correlation coefficient between daily mask CH_4_ concentration and the corresponding GreenFeed CH_4_ production averaged over Days 0 to +2 were calculated separately.

## 3. Results and Discussion

### 3.1. Association Between Mask CH_4_ Concentration and GreenFeed CH_4_ Production

The mean within-day CV among the three 5 min mask CH_4_ collections was 29.7%, indicating appreciable within-day variability among individual mask measurements. Associations between mask CH_4_ concentration and GreenFeed CH_4_ production were evaluated using individual GreenFeed days from Day −2 to Day +2 relative to mask sampling and three contiguous 3 d averaging windows (Table 3).

Correlation coefficients varied according to the temporal window considered. Among individual days, Pearson correlation coefficients ranged from 0.358 to 0.637 and Spearman correlation coefficients from 0.378 to 0.554. Although Day +2 showed the highest Pearson correlation among individual days (r = 0.637), a three-day GreenFeed average was preferred over a single-day estimate to provide a short-term CH_4_ production estimate based on a greater number of GreenFeed visits. Among the three-day windows, Days 0 to +2 showed the highest Pearson and Spearman correlations (r = 0.616 and ρ = 0.606, respectively; both *p* < 0.001) and was therefore retained for subsequent exploratory analyses.

Mask CH_4_ concentration was positively associated with GreenFeed CH_4_ production averaged over the day of mask sampling and the following two days (Days 0 to +2; Figure 2).

Pearson’s correlation analysis showed a positive association between the two measurements (r = 0.616, *p* < 0.001), which was also supported by Spearman’s rank correlation (ρ = 0.606, *p* < 0.001; *n* = 60). The significant Spearman correlation further indicates a moderate positive association in the relative ranking of observations between the two measurement approaches. When repeated observations within cows were accounted for using a linear mixed-effects model with cow included as a random intercept, mask CH_4_ concentration remained positively associated with GreenFeed CH_4_ production (β = 53.1 ± 15.7 g/day per 0.1 percentage-point increase in mask CH_4_, 95% CI: 22.3 to 84.0; *p* < 0.001). When measurements were averaged within cow to obtain one observation per animal (*n* = 32), a significant positive association between mask CH_4_ concentration and GreenFeed CH_4_ production was also observed at the cow level (Figure 3).

Pearson’s correlation coefficient was 0.536 (*p* = 0.002), while Spearman’s rank correlation coefficient was 0.505 (*p* = 0.003).

The relationship observed in the present study supports the use of exhaled CH_4_ concentration as an indicator of differences in methane emission among cows. Similar results have been reported for other concentration-based approaches. Difford et al. [8] found an individual-level correlation of 0.75 between CH_4_ concentration measured by sniffers and CH_4_ production measured in respiration chambers, compared with 0.77 after conversion of sniffer measurements to predicted CH_4_ production. Sorg et al. [9] similarly reported a repeated-measures correlation of 0.66 between CH_4_ concentration measured using a laser methane detector and GreenFeed CH_4_ production. These findings indicate that direct measurements of breath CH_4_ concentration can retain information useful for discriminating animals according to methane production.

This interpretation is also supported by van Breukelen et al. [10], who reported phenotypic correlations of 0.37–0.39 and genetic correlations of 0.71–0.76 between CH_4_ concentration measured by sniffers and CH_4_ production measured by GreenFeed. Therefore, although concentration-based measurements do not directly quantify daily CH_4_ production, they may provide a useful phenotype for identifying relatively low- and high-emitting animals. In the present study, the significant cow-level Spearman correlation (ρ = 0.505, *p* = 0.003) indicates a moderate positive association in the relative ranking of cows between the two measurement approaches.

The association between methods is likely also influenced by the short duration of mask sampling and the temporal variability of CH_4_ emission. Oss et al. [11] reported strong relationships between face-mask measurements and respiration chambers or SF_6_, but also greater day-to-day variability for the face-mask technique. Consequently, the present mask system should primarily be considered as a tool for methane phenotyping and ranking rather than for direct quantification of absolute daily CH_4_ production.

### 3.2. Within-Cow Performance of the Mask System

Four cows were repeatedly sampled on five occasions, providing a total of 20 paired observations (Figure 4).

Positive within-cow correlations between mask CH_4_ concentration and GreenFeed CH_4_ production were observed in all four cows, although their magnitude varied considerably among animals (Pearson r = 0.157–0.699). None of the individual correlations reached statistical significance (*p* > 0.05).

Spearman correlation coefficients ranged from 0.10 to 0.40, and all were non-significant (*p* > 0.05).

The lower consistency of the within-cow relationships compared with the overall between-animal analysis likely reflects the smaller range of variation occurring within the same animal and the dynamic nature of enteric CH_4_ emission. Short-term CH_4_ measurements are influenced by feeding behaviour, rumen fermentation, respiration, and eructation, and are therefore sensitive to the timing of sampling [14]. GreenFeed estimates are also affected by the number and temporal distribution of valid visits during the day [7,15]. Consequently, the two methods may not capture exactly the same short-term variation on a given day.

Despite the positive within-cow correlations observed, their lack of statistical significance, the limited number of repeated observations per animal, and the partial overlap of the GreenFeed averaging windows between consecutive sampling days, which introduced some dependence among observations, prevent a robust assessment of the ability of the mask to capture temporal variation in CH_4_ emission within cows. Further studies with a larger number of repeated measurements per animal are therefore needed to evaluate this aspect.

## 4. Conclusions

The positive association between CH_4_ concentration measured with the mask system and CH_4_ production recorded by GreenFeed indicates that the proposed method can provide information on differences in methane production among dairy cows. In particular, the rank-based association observed at the cow level suggests that mask CH_4_ concentration may have potential as a screening phenotype, although further studies are needed to establish its ability to reliably rank individual cows according to their methane emissions. The exploratory within-cow analysis showed positive but non-significant relationships in the four repeatedly measured animals although further studies with larger numbers of cows and repeated measurements are required to confirm this finding. Overall, the mask system represents a simple approach with potential application for methane phenotyping and ranking of dairy cows according to their relative methane emissions.

## Figures and Tables

**Figure 1:**
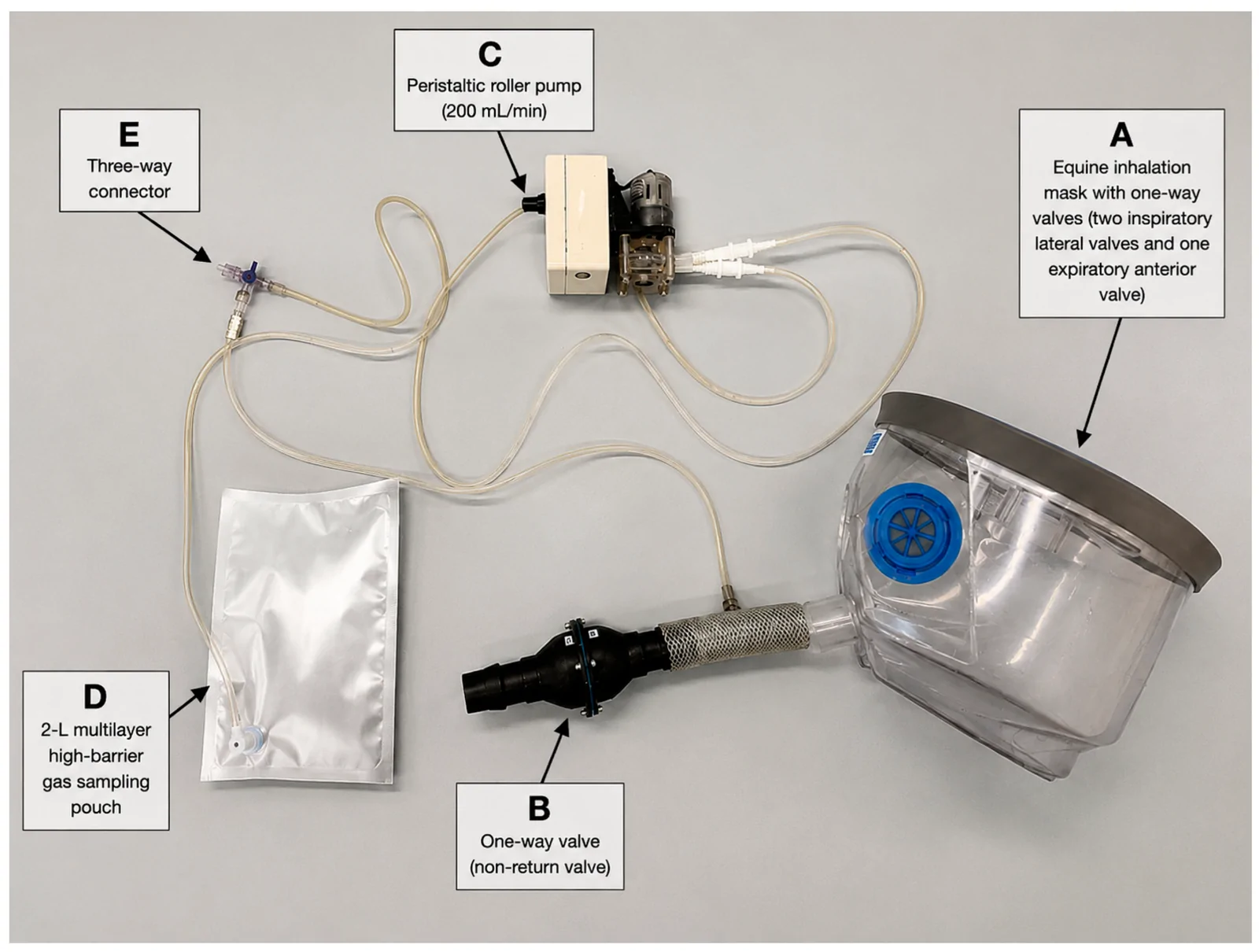
Modified mask-based system used for collection of exhaled breath from dairy cows. The system consisted of (**A**) an equine inhalation mask equipped with two lateral inspiratory one-way valves and one anterior expiratory one-way valve; (**B**) an expiratory one-way (non-return) valve; (**C**) a peristaltic roller pump operating at a constant flow rate of 200 mL/min; (**D**) a 2 L multilayer high-barrier gas sampling pouch; and (**E**) a three-way connector.

**Figure 2:**
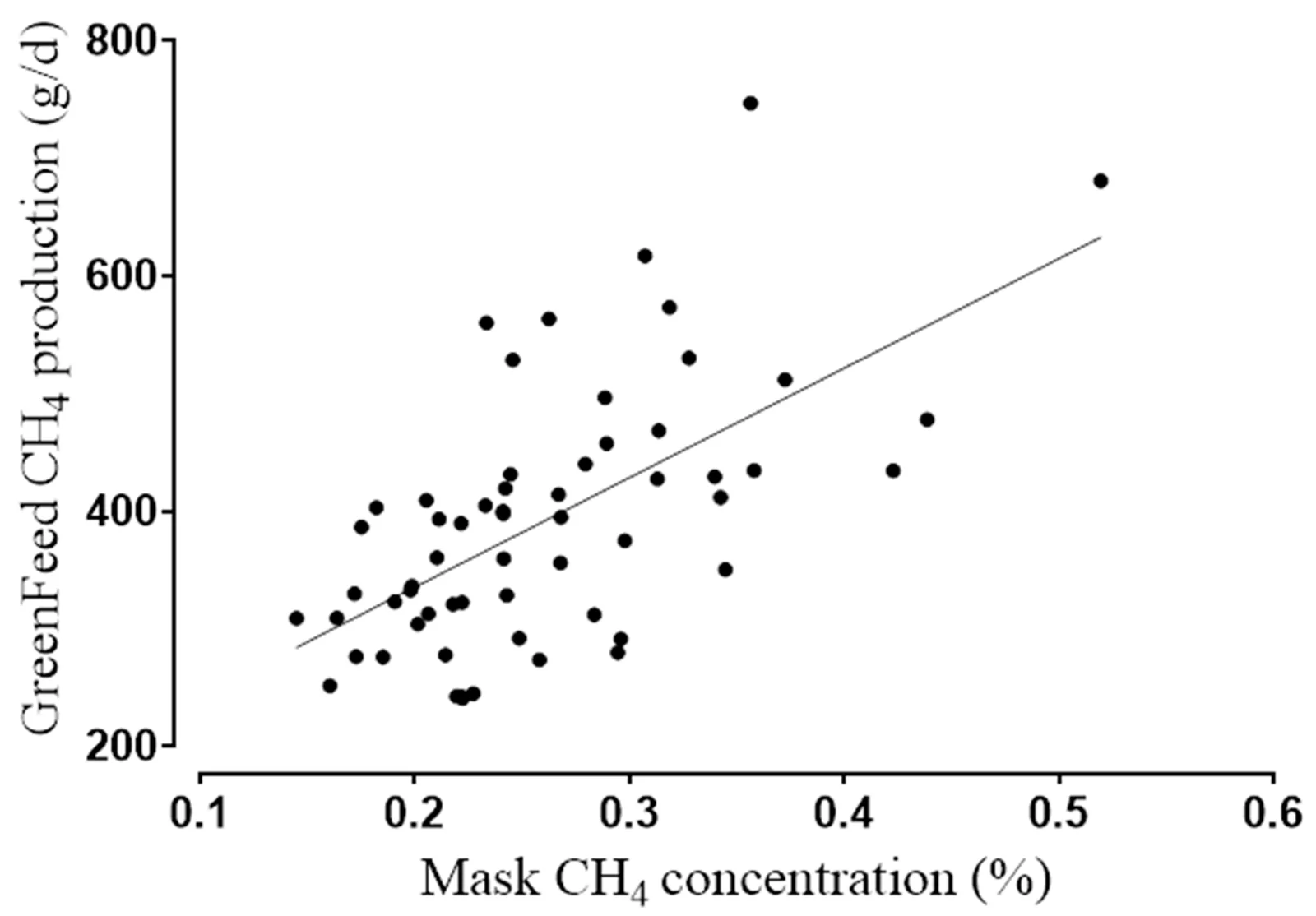
Relationship between CH_4_ concentration measured using the mask-based sampling system and GreenFeed CH_4_ production averaged over the day of mask sampling (Day 0) and the following two days (Days +1 and +2) in lactating dairy cows (*n* = 60 observations). The solid line represents the least-squares line of best fit across the 60 observations. Pearson’s correlation was r = 0.616 (*p* < 0.001), and Spearman’s rank correlation was ρ = 0.606 (*p* < 0.001).

**Figure 3:**
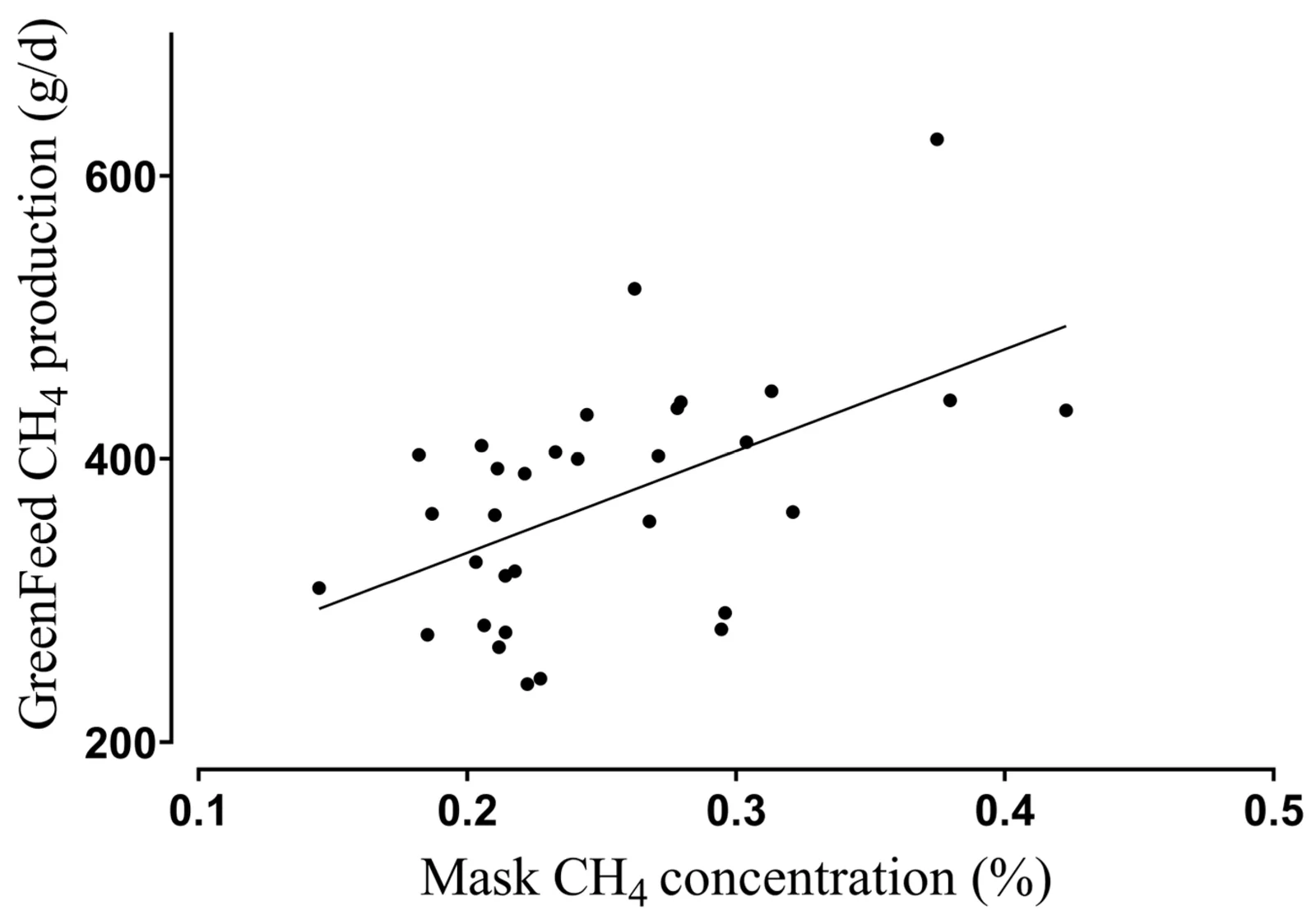
Cow-level relationship between mean CH_4_ concentration measured using the mask-based sampling system and mean GreenFeed CH_4_ production in lactating dairy cows (*n* = 32). For each cow, values were averaged across all available sampling occasions, with GreenFeed CH_4_ production calculated over Days 0 to +2 relative to each mask sampling day. Each point represents one cow. The solid line represents the least-squares line of best fit. Pearson’s correlation was r = 0.536 (*p* = 0.002), and Spearman’s rank correlation was ρ = 0.505 (*p* = 0.003).

**Figure 4:**
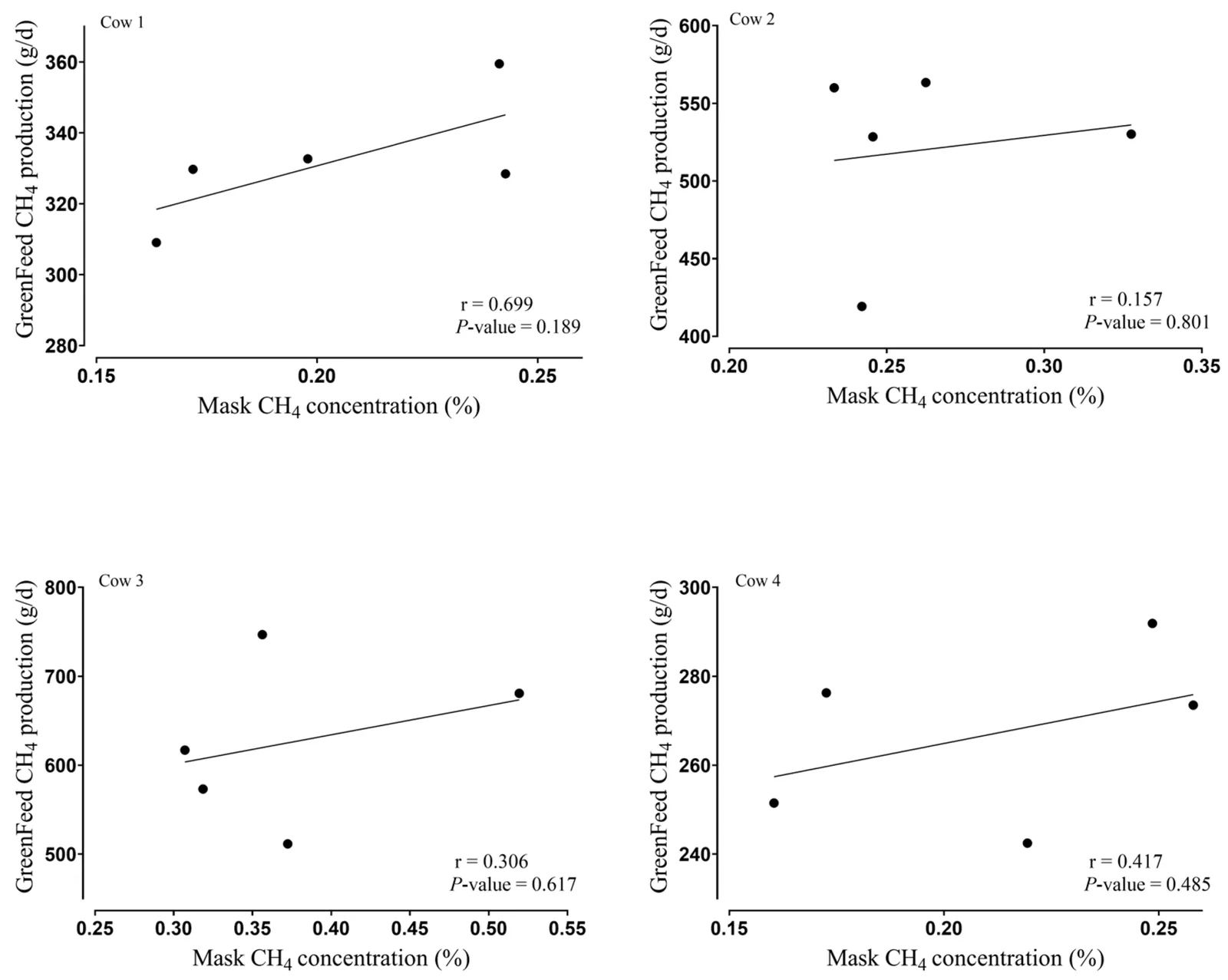
Within-cow relationships between CH_4_ concentration measured using the mask-based sampling system and GreenFeed CH_4_ production averaged over the day of mask sampling (Day 0) and the following two days (Days +1 and +2) in four lactating dairy cows repeatedly sampled on five occasions. Panels represent individual cows. Each point represents a paired daily observation, and solid lines represent the least-squares lines of best fit. Pearson correlation coefficients (r) and corresponding *p*-values are reported within each panel.

**Table 1:** Descriptive characteristics of the lactating dairy cows included in the study (*n* = 32).

Variable	Mean ± SD
Age, months	48.1 ± 16.0
Days in milk, day	198 ± 68
Body weight, kg	636 ± 74
Body condition score	2.76 ± 0.35
Milk yield, kg/day	36.2 ± 6.3
TMR dry matter intake, kg DM/day	25.8 ± 2.6
GreenFeed bait intake, kg DM/day	1.11 ± 0.27
Total dry matter intake, kg DM/day	26.8 ± 2.5

**Table 2:** Ingredient and nutrient composition (% of DM unless otherwise noted) of TMR and GreenFeed bait pellets.

Item	TMR	GreenFeed Bait Pellets ^4^
Ingredients		
Corn silage	20.0	
Concentrate mix ^1^	21.9	
Alfalfa hay	14.6	
Wheat bran	14.6	
Oat silage	8.9	
Soybean meal	10.9	
Sunflower meal	3.3	
Beet molasses	3.0	
Hydrogenated fats	0.8	
Vitamin and mineral premix ^2^	2.0	
Nutrient composition, % of DM ^3^		
Starch	24.1	
CP	16.3	22.2
Metabolizable protein (MP)	10.0	
Lysine (% MP)	6.57	
Methionine (% MP)	2.15	
NDF	32.4	
Crude fat (EE)	2.9	5.0
Crude fiber		6.1
Ash		5.0
Ca	0.8	
P	0.5	
Mg	0.3	
Na	0.3	0.06
Energy, Mcal/kg of DM		
ME	2.6	
NE_L_	1.7	

^1^ Concentrate mix contained 60% corn flour, 40% rolled barley. ^2^ Vitamin and mineral mix provided 450,000 IU of vitamin A, 45,000 IU of vitamin D, 900 mg of vitamin E, 35 mg of vitamin K, 42 mg of vitamin B1, 0.6 mg of vitamin B12, 6000 mg of vitamin PP, 300 mg of Mn, 1030 mg of Zn, 80 mg of Cu, and 15 mg of I, and 2.7 mg Se. ^3^ The DM was 52.3% for TMR; for the GreenFeed bait pellets was 90%. ^4^ Tekno Robot CREI (Ferrero Mangimi S.p.A., Farigliano, Cuneo, Italy).

**Table 3:** Associations between mask CH_4_ concentration and GreenFeed CH_4_ production calculated using different temporal windows relative to the day of mask sampling. Day 0 indicates the day of mask sampling; negative and positive values indicate days before and after mask sampling, respectively. Three-day windows represent the arithmetic mean of GreenFeed CH_4_ production across the indicated consecutive days. The Days 0 to +2 window was selected for the subsequent analyses.

GreenFeed Temporal Window	Days Included	*n*	Pearson r	*p*-Value	Spearman ρ	*p*-Value
Day −2	−2	60	0.394	0.002	0.378	0.003
Day −1	−1	60	0.358	0.005	0.424	<0.001
Day 0	0	60	0.539	<0.001	0.529	<0.001
Day +1	+1	60	0.439	<0.001	0.440	<0.001
Day +2	+2	60	0.637	<0.001	0.554	<0.001
Days −2 to 0	−2, −1, 0	60	0.494	<0.001	0.519	<0.001
Days −1 to +1	−1, 0, +1	60	0.528	<0.001	0.541	<0.001
Days 0 to +2	0, +1, +2	60	0.616	<0.001	0.606	<0.001

## Data Availability

The raw data supporting the conclusions of this article will be made available by the authors upon request.

## Abbreviations

The following abbreviations are used in this manuscript:

ADF	Acid detergent fiber
CH_4_	Methane
CO_2_	Carbon dioxide
CP	Crude protein
DM	Dry matter
EE	Ether extract
GF	GreenFeed
GC	Gas chromatography
ME	Metabolizable energy
MP	Metabolizable protein
NDF	Neutral detergent fiber
NEL	Net energy for lactation
NEM	Net energy for maintenance
RC	Respiration chamber
